# Systematic Review and Meta-Analysis of Individual Patient Data to Assess the Sensitivity of Cervical Cytology for Diagnosis of Cervical Cancer in Low- and Middle-Income Countries

**DOI:** 10.1200/JGO.2016.008011

**Published:** 2017-03-01

**Authors:** Alejandra Castanon, Rebecca Landy, Dimitrios Michalopoulos, Roshni Bhudia, Hannah Leaver, You Lin Qiao, Fanghui Zhao, Peter Sasieni

**Affiliations:** **Alejandra** **Castanon,** **Rebecca Landy, Dimitrios Michalopoulos, Roshni Bhudia, Hannah Leaver,** and **Peter Sasieni,** Wolfson Institute of Preventive Medicine, London, United Kingdom; and **You Lin Qiao** and **Fanghui Zhao,** National Cancer Center and Cancer Hospital, Chinese Academy of Medical Sciences, Beijing, China.

## Abstract

**Purpose:**

To assess the sensitivity of cervical cytology to cancer by pooling individual patient cytology results from cancers diagnosed in studies that assessed cervical screening in low- and middle-income countries.

**Methods:**

Two authors reviewed studies identified through PubMed and Embase databases. We included studies that reported cervical cytology in which at least one woman was diagnosed with cervical cancer and in which abnormal cytology results were investigated at colposcopy and through a histologic sample (if appropriate). When cytology results were not reported in the manuscript, authors were contacted. Stratified analyses and meta-regression were performed to assess sources of heterogeneity between studies.

**Results:**

We included 717 cancers from 23 studies. The pooled sensitivity of cytology to cancer at a cutoff of a high-grade squamous intraepithelial lesion (HSIL) or worse was 79.4% (95% CI, 67.7% to 86.0%). Results from stratified analyses did not differ significantly, except among studies that recruited symptomatic women or women referred because of abnormal cytology, when the sensitivity of cytology was much higher (95.9%; 95% CI, 86.5% to 99.9%). The cutoff of an HSIL or worse detected 85% of the cancers that would have been detected at a cutoff of atypical squamous cells of undetermined significance or worse (relative sensitivity, 85.2%; 95% CI, 80.7% to 89.7%).

**Conclusion:**

Cytology at a high cutoff could be an excellent tool for targeted screening of populations at high risk of cervical cancer with a view to diagnose cancer at an earlier stage.

## INTRODUCTION

Cervical cancer is the fourth most common cancer in women. Approximately 85% of the global burden occurs in less-developed regions.^1^ To reduce the incidence of cervical cancer, screening has been offered to women in an attempt to identify precursors that can be treated to avoid progression to cancer. Cervical cytology relies on the ability of sample takers to sample the affected region adequately and on the ability of observers to identify precursor disease. In less-developed countries, the lack of both infrastructure and quality management has led to wide variations in the sensitivity and specificity of cytology testing. In a recent meta-analysis^2^ that compared screening methods in low-income countries, the sensitivity of cervical cytology to cervical intraepithelial neoplasia (CIN) grade 2 or worse (CIN2+) in studies that assessed visual inspection with acetic acid and cytology ranged from 33% to 100%. This wide variation in cytology performance has meant that recent research has focused on new screening technologies (eg, human papillomavirus [HPV] testing), which are less user dependent.

In resource-poor settings with limited facilities to treat advanced cancers, the ability to use cytology in targeted high-risk populations as a tool to detect cancer at an early stage could have a big impact on cervical cancer mortality. However, little has been published on the sensitivity of cytology to cancer, and strategies to detect cervical cancer at an early stage have been overlooked.

Here, we aim to assess the sensitivity of cytology to cancer by pooling cytology results from cancers diagnosed among participants in studies that assessed cytology screening in low- and middle-income countries (LMICs).

## METHODS

### Inclusion Criteria and Outcomes

The protocol outlined the research question, populations, exposures, outcome of interest, search strategies, study selection, inclusion and exclusion criteria, and methods for data extraction and statistical analysis (including subgroup analyses but not the meta-regression).

We searched the PubMed and Embase databases with standard terms to cover the concepts of cervical intraepithelial neoplasia, sensitivity, Pap test and developing countries (see Data Supplement for the full search description). We searched for published articles that resulted from identified conference abstracts. In addition, bibliographies of published papers were searched to locate additional papers. A few manuscripts were identified after authors were contacted about related studies. We identified studies in English published through December 2014 that included cytology, were conducted in LMICs (as determined by the World Bank list of economies, July 2014),^3^ and in which at least one woman was diagnosed with invasive cervical cancer.

All studies were reviewed by two investigators independently (divided among A.C., R.L., D.M., H.L., and R.B.) for eligibility criteria according to a standardized inclusion form. Any differences of opinion were reconciled by a consensus between A.C. and R.L.

Inclusion criteria were studies that reported cervical cytology and confirmed abnormal results at colposcopy and through a histologic sample (if appropriate). Studies were eligible even if the cytology results were not reported in the manuscript. We excluded studies restricted to HIV-positive women, studies of women who had all previously undergone cervical treatment, and studies that were restricted to women who had a single cytology result (eg, only atypical squamous cells of undetermined significance [ASCUS] cytology). Most cytology results were reported with the Bethesda system terminology; however, a few studies used CIN terminology. We classified results in risk order as follows: normal; inadequate; ASCUS; mild dysplasia/CIN grade 1 grouped with low-grade squamous intraepithelial lesions (LSIL); atypical glandular cells grouped with atypical squamous cells unable to exclude high-grade squamous intraepithelial lesions (HSIL; ASC-H); moderate dysplasia/CIN grade 2, severe dysplasia/CIN grade 3, carcinoma in situ, and adenocarcinoma in situ grouped with HSIL; and squamous and adenocarcinoma grouped as invasive cancers.

Two studies reported both conventional and liquid-based cytology (LBC).^4,5^ However, the LBC was reported by experts, so conventional cytology results were considered in the main analysis. As a subanalysis, we show LBC results from these two studies and from those in Zhao et al.^6^

### Data Collection Process

When data were not reported in the required format in the published manuscript, we attempted to contact the corresponding author from each study via e-mail. Two reminders were sent during a period of 8 months and/or alternative authors were contacted.

We collected information on the cytology results and number of cancers by asking the authors to complete a simple table of aggregated data (Data Supplement). Results reported in the manuscript were extracted directly.

Information was extracted from each included study on the following: study population data, including country, age, and inclusion and exclusion criteria; study design, including type of screening tests offered, population enrolled, and criteria for assessment of disease; number of women tested with cytology overall and with a cancer diagnosis; type of cytology laboratory used; and cytology results from the last test before cancer diagnosis regardless of how long before diagnosis.

Two authors assessed the quality of included studies through the QUADAS-2 tool^7^ for quality assessment of diagnostic accuracy studies. Disagreements were resolved through discussion.

### Summary Measures and Data Analysis

Sensitivity was calculated as the proportion of women with cancer who had a positive test when ASCUS or worse, LSIL or worse, and HSIL or worse were considered. Exact binomial 95% CIs were calculated (and, when the sensitivity was 100% or 0%, we estimated 97.5% one-sided intervals). We performed a variance-stabilizing transformation by taking the arcsine of the square root of the sensitivity estimate and 1 ÷ (4 × the number of cancers) as the variance.^8^ These were analyzed in STATA 12 with the METAAN command (StataCorp, College Station, TX). The pooled estimates (and 95% CIs) were back-transformed to give the sensitivity.

We assessed statistical heterogeneity with the Cochran *Q* and Higgins *I*^2^ tests, and we defined heterogeneity as *I*^2^ > 25% or *P* < .05. In addition, meta-regressions were run as separate univariate analyses to estimate how much of the heterogeneity was explained by covariates.^9,10^

Subanalyses and meta-regressions were conducted by pooling results from studies on the basis of the following: criteria for assessment of disease—all enrolled women were referred for colposcopy assessment, or women who tested positive to any screening test were referred to colposcopy; type of population studied—symptomatic women and those referred after an abnormal cytology test, or a screening population; quality of cytopathology—local laboratory without mention of special training for the study (lower quality), or a cancer referral center cytology laboratory or training and quality assurance carried out as part of the study (higher quality); number of cancers in each study—one to nine cancers (small), 10 to 24 cancers (medium), or 25 or more cancers (large); World Bank developmental indicator—LMIC (no studies in low-income countries), or upper-middle income country; and type of screening test offered— HPV testing, no HPV testing, or LBC.

To explore how the quality of cytology affects the sensitivity of the test, we included as a continuous variable in the meta-regression the sensitivity of cytology to CIN2+ at a cutoff of an ASCUS or worse, when available.^4,11-23^

### Details of Ethics Approval

The study used a combination of previously published data and aggregated data from individual studies. All data were anonymous. No ethical approval was required.

## RESULTS

**Fig 1:**
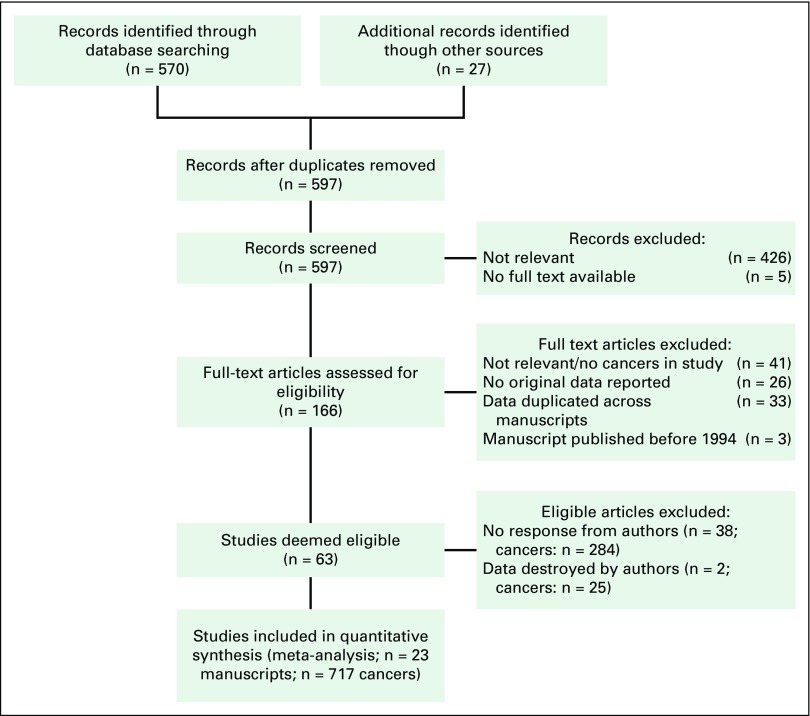
Flowchart of located studies.

A total of 570 unique studies were identified through PubMed and Embase. An additional 27 studies were identified through searches of reference lists. Of the 597 abstracts reviewed, 426 (71%) were excluded. Full texts were reviewed for 166 papers; we were unable to locate five papers. We excluded 26 manuscripts with no original data, 41 that were not relevant or did not contain any cervical cancers, and 33 because of duplication of data across more than one manuscript. Three manuscripts were published before 1994, and, although we attempted unsuccessfully to contact the authors, we considered it unlikely that research data would have been kept for longer than 20 years; therefore, we excluded these manuscripts. A total of 63 manuscripts were deemed eligible (Fig 1).

Data were reported in the format required in 11 studies, which included a total of 247 cervical cancers.^11,14,16,18,19,21,23-27^ We attempted to contact the investigators from the remaining 52 studies. Authors responded with data for 12 separate studies.^4-6,13,15,17,20,22,28-31a^ For 10 studies, contact details were out of date or not provided. No data were available for two of the requested manuscripts, and no response was obtained from 28 authors. Approximately 309 cancers were included among the 40 studies for which no response was obtained (the number of cancers were not reported in seven studies).

**Fig 2:**
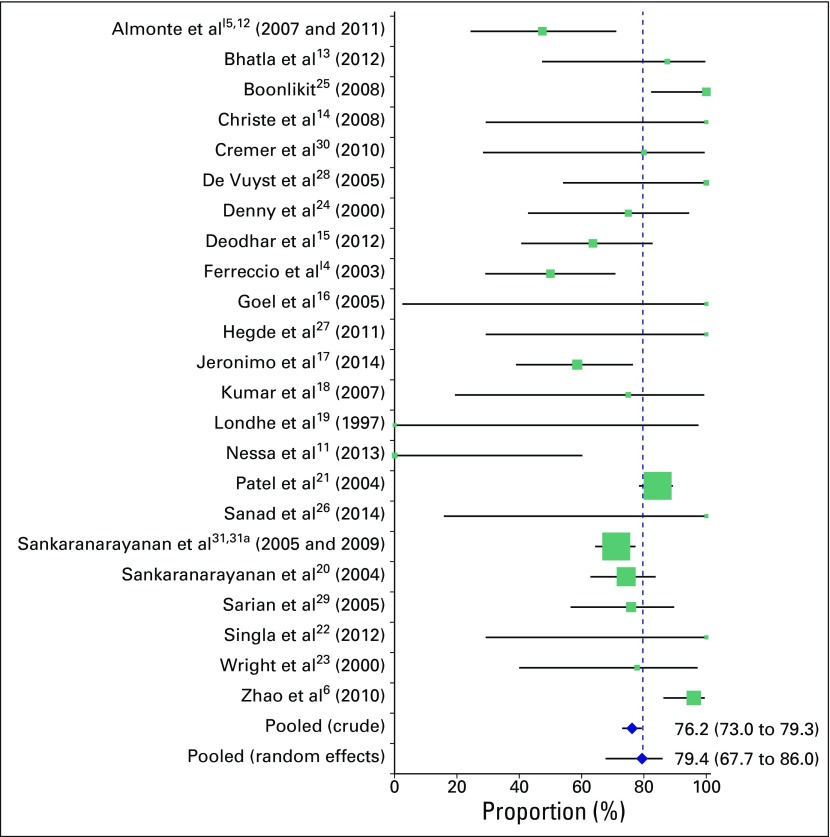
Sensitivity (percent and 95% CI) of cytology to cancer at a cutoff of high-grade squamous intraepithelial lesion or worse. The center of the square provides the value for the sensitivity, and the size represents the number of cancers included in each study.

For analysis, we include 23 studies with 717 cancers (Table 1). We estimate that we included 70% of all cancers from the identified literature. Sensitivity results for individual studies at a cutoff of HSIL or worse are shown in Figure 2. A summary of cytology results and crude sensitivities for included studies is listed in Table 2. The crude pooled analysis of all studies showed a sensitivity of 76.2% (95% CI, 73.0% to 79.3%) of cervical cytology to cancer at a cutoff of HSIL or worse. The random effects model estimated the sensitivity to be 79.4% (95% CI, 67.7% to 86.0%), and substantial heterogeneity between studies was observed (*I*^2^, 88.8%; *P* < .001).The respective results for a cutoff of LSIL or worse were 80.9% (95% CI, 78.0% to 83.8%) and 86.3% (95% CI, 75.2% to 94.5%); for a cutoff of ASCUS or worse, they were 87.6% (95% CI, 85.2% to 90.0%) and 91.1% (95% CI, 81.2% to 97.5%).

**Table 1:**
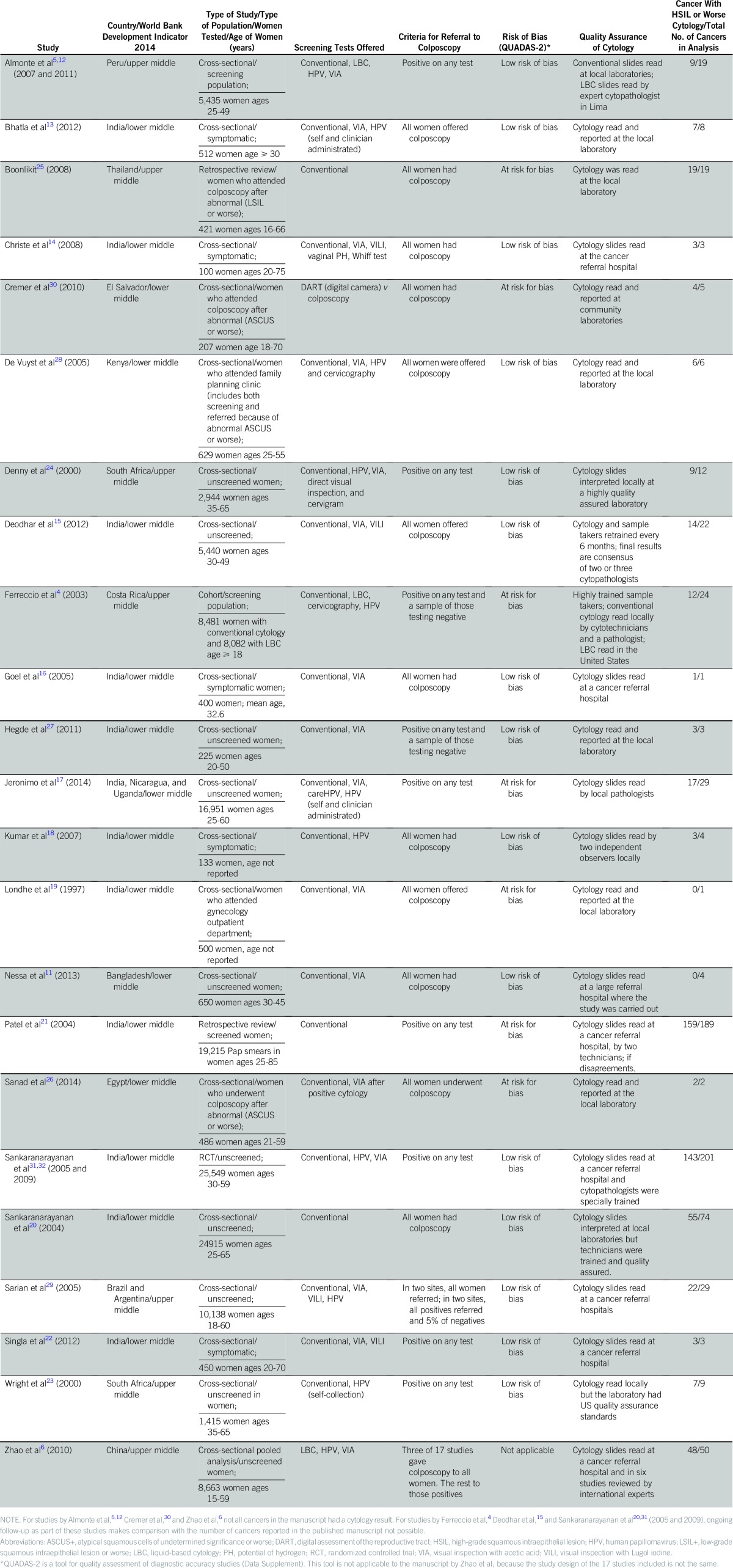
Summary of Included Studies

**Table 2:**
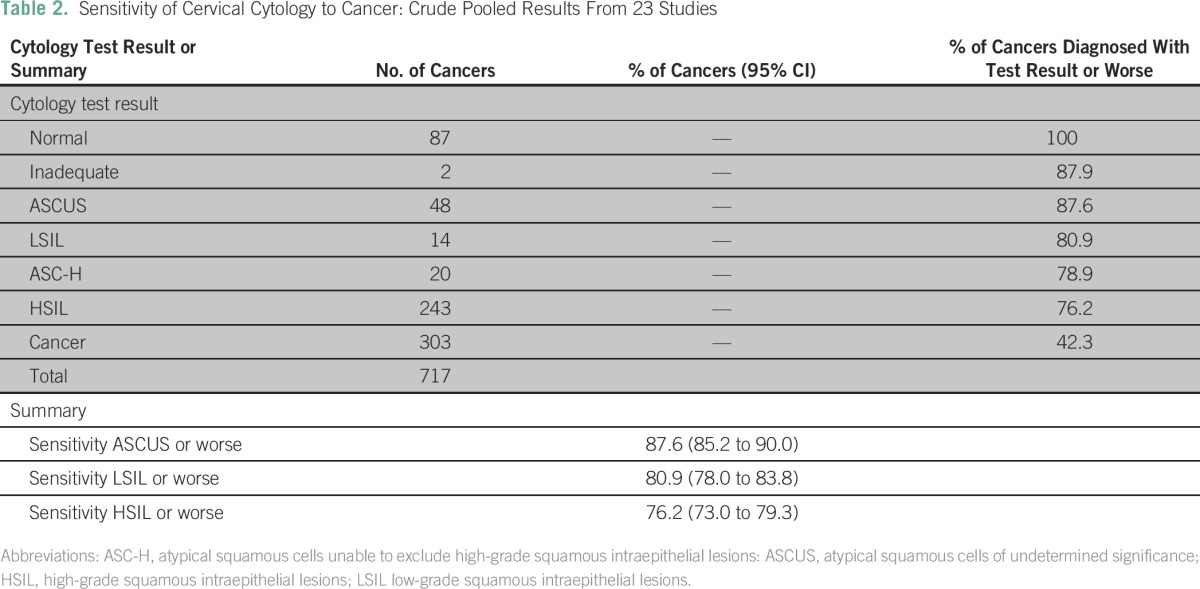
Sensitivity of Cervical Cytology to Cancer: Crude Pooled Results From 23 Studies

**Fig 3:**
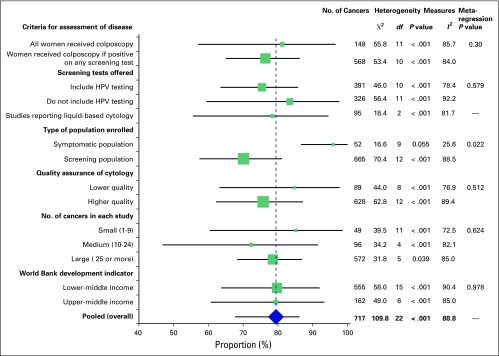
Pooled analysis and subanalysis of the sensitivity of cytology to cancer at a cutoff of high-grade squamous intraepithelial lesion or worse. The center of the square provides the value for the sensitivity, and the size represents the number of cancers included in each study.

Quality assessment of included studies is listed in Table 1 (Data Supplement). The majority of studies (n = 15) were deemed at low risk of bias. Bias was assessed through several subanalyses, which are presented at a cutoff of HSIL or worse (Table 3; Fig 3).

**Table 3:**
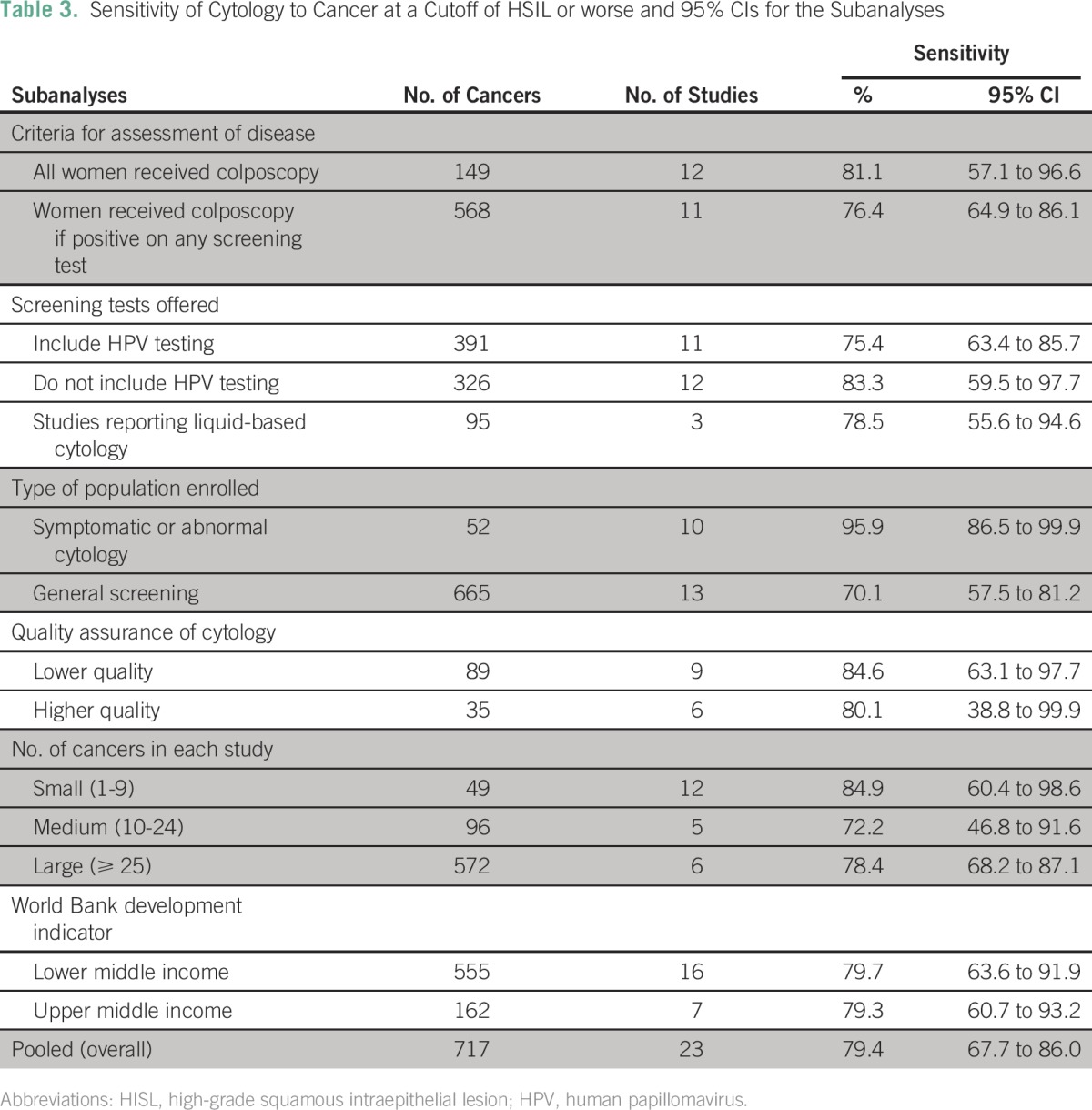
Sensitivity of Cytology to Cancer at a Cutoff of HSIL or worse and 95% CIs for the Subanalyses

Sensitivity was 81.1% (95% CI, 57.1% to 96.6%) among studies that assessed disease status on all enrolled women compared with 76.4% (95% CI, 64.9% to 86.1%) among studies that only assessed it in women who tested positive to any of the screening tests offered. Note that, in some studies, cytology was the only screening test, but in all studies, women with any abnormality on cytology were referred to colposcopy. There was evidence of statistical heterogeneity among studies in both analyses (all enrolled women: *I*^2^, 85.7%; *P* < .001; only women who tested positive: *I*^2^, 84.0%; *P* < .001).

The effect of verification bias on the sensitivity of the test was studied by splitting the studies into those that included HPV testing and those that did not, because a large proportion of women who are negative on cytology are referred to colposcopy when HPV testing also is carried out. As predicted, we observe lower sensitivities when HPV testing was used to ascertain disease status—75.4% (95% CI, 63.4% to 85.7%)—compared with 83.3% (95% CI, 59.5% to 97.7%) among studies that did not include it.

When the LBC results, instead of the conventional cytology results, for Almonte et al^5,12^ and Ferreccio et al^4^ were included, the overall sensitivity of the test was higher (Data Supplement), because the LBC results had better sensitivity. However, when results from Zhao et al^6^ (the only other study to report LBC) were added, the sensitivity of LBC was similar to the overall pooled estimate (78.5%; 95% CI, 55.6% to 94.6%).

Among studies that provided cytology testing to the general screening population, the sensitivity was 70.1% (95% CI, 57.5% to 81.2%) compared with 95.9% (95% CI, 86.5% to 99.9%) among studies that recruited symptomatic women or women referred because of a previous abnormal cytology. Little evidence of heterogeneity among studies that included women with symptoms or those referred because of abnormal cytology was observed (*I*^2^, 25.6%; *P* = .06). These results are supported by the meta-regression: 76% of the variance was between studies (*I*^2^ residual, 75.9%; *P* = .022). The population enrolled explained 43% of the variance between studies, and 57% remained unexplained (adjusted *R*^2^, 43.0%). Meta-regression analyses did not show a significant effect of any of the other covariates (Fig 3).

When the quality of the cytology was considered, we found higher sensitivities, although CI overlap, among studies that had lower-quality cytology (84.6%; 95% CI, 63.1% to 97.7%) than higher-quality cytology (75.8%; 95% CI, 62.1% to 87.2%). Similar results were observed when the number of cancers in each study was considered. It is worth noting that studies with lower-quality cytology only were also more likely to offer colposcopy to women with abnormal cytology. The World Bank development indicator made little difference to the sensitivities (Table 3).

We estimated the relative sensitivity of HSIL or worse compared with ASCUS or worse to account for the exclusion of women with negative cytology from some studies. For this analysis, the study by Boonlikit^25^ was excluded, because no one with a result of ASCUS was enrolled. The relative sensitivity was 85.2% (95% CI, 80.7% to 89.7%). There was no evidence of statistical heterogeneity between studies in this analysis (*I*^2^, 6.2%, *P* = .730).

The sensitivity of cytology to cancer was strongly correlated to the sensitivity of cytology to CIN2+; 46% of the variance was between studies (*I*^2^ res, 45.9%; *P* = .005). Sensitivity to CIN2+ was able to explain 85% of the variance between studies, and only 15% remained unexplained (adjusted *R*^2^, 85.0%).

## DISCUSSION

Overall, we found that cytology at a cutoff of HSIL or worse had a sensitivity to cancer of 79%. Considerably higher sensitivity (96%) was observed among studies that included symptomatic women or women who had abnormal cytology than among studies that enrolled women from the general screening population (70%). We consider a cutoff of HSIL or worse to be appropriate when cytology was used to diagnose cancer, because it detected 85% of cancers with abnormal cytology. Results suggest that the use of cytology to identify cancer would be well suited for use in high-risk or targeted groups.

This study takes data from studies that use cervical cytology as a screening tool and assessed its use as a test for early detection of cancer. It is the first study, to our knowledge, to evaluate the use of cytology to diagnose cervical cancer in LMICs, and it includes approximately 70% of cancers identified as eligible for this study from a wide range of settings.

Verification bias could potentially affect sensitivity of cytology in all included studies, because colposcopy can easily miss endocervical cancers, particularly when it is not guided by prior cytology. Here, we take a pragmatic approach and consider verification bias to be minimal if all HPV-positive women have colposcopy. The risk of bias, then, will be related to the proportion of those referred to colposcopy who receive colposcopy. It is seen that the absolute sensitivity of cytology at HSIL or worse is indeed dependent on the study population and referral criteria, whereas the relative sensitivity (compared with ASCUS or worse) is homogeneous.

Authors from research organizations that mainly aim to carry out this type of research were more likely to respond to our requests for data, and cytology samples taken as part of these studies may be better than cytology taken in routine settings.

Judgement of the quality of cytology through the details reported in each study was not straightforward and is subjective. Bias toward a higher sensitivity than that observed in routine practice may remain.

We used the country income level from the World Bank in 2014, though most of the studies were conducted before then. It is possible that countries have moved from lower-middle to upper-middle income levels, or vice versa, in the intervening period; this would lead to misclassification bias in that subanalysis.

Most cross-sectional studies took the cytology within 3 months of the diagnosis of cancer. However, for some studies, in particular the cohort studies, we do not know how long before diagnosis of cancer the cytology was taken. One would expect the sensitivity of the test for cancer to be lower, the longer it was before diagnosis.

Despite these limitations, the overall high sensitivity of HSIL or worse cytology to invasive cancer is clear.

The sensitivity of cytology to cancer at a cutoff of HSIL or worse (79%) was similar to the sensitivity of cytology at a cutoff of ASCUS or worse to CIN2+ reported in a meta-analysis by Mustafa et al.^2^ They found that, among studies (all of which were from LMICs) that compared visual inspection with acetic acid to cytology, the sensitivity of cytology to CIN2+ at a cutoff of ASCUS or worse was 84% (95% CI, 76% to 90%). This suggests that the sensitivity of cytology to cancer is similar to its sensitivity to CIN2+ in a population screening context.

The main benefit of using cytology at a high cutoff to diagnose cervical cancer would be earlier stage at diagnosis, with the ability to offer lifesaving treatment options, reduce mortality, and improve quality of life. In developing countries, which lack screening programs, the incidence of cervical cancer may be up to six times higher than in developed countries, and up to 80% of patients present with advanced disease.^32^ In addition, facilities to treat advanced cancers are limited in many developing countries; for example, many countries have more than 2 million people per radiotherapy unit, and some countries do not have any radiotherapy units.^33^

The use of cytology to downstage cancers has not been given appropriate consideration as a viable alternative, even though the low-cost alternative (visual inspection of the cervix with a speculum) is proven not to be a suitable primary screening modality for cervical cancer.^34^ In England, Landy et al^35^ found that the majority of cancers (72.6%) in women diagnosed at age 66 years or older who did not have a cytology test within 12 months of diagnosis were diagnosed with FIGO stage 2 or worse. However, among women of the same age who had cytology in the 12 months before diagnosis (presumably because of symptoms, because screening is not offered in this age group), the proportion with FIGO stage 2 or worse disease decreased to 56.2%, and these women had better survival than women without cytology. Several other authors also have found that, among symptomatic woman, diagnosis of cervical cancer through cytology resulted in better survival.^36,37^

Although one may expect the sensitivity of cytology to cancer to be high even when the sensitivity to CIN2+ is low, we found that the sensitivity of cytology to cancer was highly related to the sensitivity to CIN2+ (at a lower cutoff). Therefore, quality control of cytology will remain necessary when cytology is used to diagnose cancer.

Restriction of cytology to symptomatic women and HSIL or worse referral for further investigation at colposcopy of those who have a result of HSIL or worse would free up resources that could be used to improve the quality of cytology to ensure sensitivities of HSIL or worse to cancer greater than 75% in all settings.

In conclusion, cytology testing at a threshold of HSIL or worse is an excellent tool for targeted screening of populations at high risk of cervical cancer, with a goal of cancer diagnosis at an earlier stage. Evidence suggests that a sensitivity of greater than 75% would be observed in all settings.
